# Impact of Stewardship Pharmacist Driven MRSA Nasal Surveillance and De-escalation of Anti-MRSA Therapy

**DOI:** 10.1017/ash.2024.156

**Published:** 2024-09-16

**Authors:** Jessica Dillon, Jessica Abrantes-Figueiredo, Dora Wiskirchen, Domenic Vita

**Affiliations:** Saint Francis Hospital and Medical Center; Trinity Health of New England

## Abstract

**Background:** Intravenous vancomycin is commonly used as initial empiric coverage for pneumonia but is often unnecessary. MRSA nasal surveillance cultures (MRSA nasal swab) have been highlighted in recent literature and the 2019 IDSA Pneumonia Treatment Guidelines as a tool to avoid unnecessary MRSA coverage in pneumonia. A negative MRSA nasal swab can be utilized by clinicians to de-escalate anti-MRSA therapies for pneumonia. The purpose of this study is to determine if implementing stewardship pharmacist driven MRSA nasal surveillance increases use of the test and reduces the inappropriate use of vancomycin for MRSA coverage in patients with pneumonia. **Method:** This study was a retrospective chart review and was approved by the Trinity Health of New England Institutional Review Board. For this initiative, a stewardship pharmacist evaluated all patients receiving vancomycin for anti-MRSA therapy at Saint Francis Hospital and Medical Center in Hartford, CT. If the patient’s indication was pneumonia and a MRSA nasal swab had not been ordered, the pharmacist contacted the patient’s provider and requested an order for it. Upon receipt of a negative MRSA nasal swab result, the pharmacist recommended discontinuation of vancomycin to the provider if appropriate. Outcomes from the first four weeks of the pharmacist-driven initiative (April 10, 2023 to May 5, 2023) were compared to the four weeks prior to the initiative (March 13, 2023 to April 7, 2023) as a control group. The primary outcome of this study was percentage of patients who received a MRSA nasal swab. Secondary outcomes included percentage of patients who had vancomycin appropriately de-escalated based on MRSA nasal swab results and mean length of vancomycin therapy. **Result:** 116 patients met inclusion criteria: 61 in the control group and 55 in the intervention group. Percentage of swabs ordered increased from 36.1% (22/61) without pharmacist intervention to 80.0% (44/55) with pharmacist intervention (p < 0 .0001). There were also increased rates of vancomycin de-escalation in patients with pharmacist intervention, with 58.2% (32/55) of patients in the intervention group having vancomycin discontinued following a negative MRSA swab compared to 19.7% (12/61) in the control group (p < 0 .0001). **Conclusion:** The results suggest implementing a pharmacist driven MRSA nasal surveillance program into practice could increase the number of MRSA nasal swabs ordered and in turn promote more timely de-escalation of vancomycin in patients with pneumonia. The results from this study can be used to support the wide-spread use of pharmacist driven MRSA nasal surveillance protocols at other institutions.

